# Comparison of verbal and computerised months backwards tests in a hospitalized older population

**DOI:** 10.1007/s40520-022-02205-w

**Published:** 2022-08-05

**Authors:** Martin Mulligan, Leona Lally, Dimitrios Adamis, David Meagher, Chris Exton, Colum Dunne, Geraldine McCarthy

**Affiliations:** 1 School of Medicine, NUIG, Galway, Ireland; 2 Department of Psychiatry, Sligo Mental Health Service, Sligo University Hospital, Sligo, Ireland; 3 Department of Psychiatry, Graduate Entry Medical School, University of Limerick, Limerick, Ireland; 4 Department of Computer Sciences, University of Limerick, Limerick, Ireland; 5 Cognitive Impairment Research Group, Graduate Entry Medical School, University of Limerick, Limerick, Ireland

**Keywords:** Delirium, Dementia, Attention, Months backwards test, Information technology

## Abstract

**Background:**

Delirium is extremely prevalent, yet underdiagnosed, in older patients and is associated with prolonged length of hospital stay and higher mortality rates. Impaired attention is the cardinal deficit in delirium and is a required feature in diagnostic criteria. The verbal months backwards test (MBT) is the most sensitive bedside test of attention, however, hospital staff occasionally have difficulty with its administration and interpretation. We hypothesise that the MBT on an electronic tablet may be easier and more consistent to use for both experienced and unexperienced medical professionals and, if the diagnostic efficacy was similar, aid delirium diagnosis.

**Aim:**

We aim to investigate the correlation of the verbal MBT with a computerised MBT application.

**Methods:**

Participants recruited (age > 65, *n* = 75) were allocated to different cohorts (Dementia and Delirium (DMDL), Dementia (DM), Delirium (DL), No Neurocognitive Disorder (NNCD)) and were administered both the verbal and electronic versions.

**Results:**

Correlation between measurements were: overall Spearman’s rho = 0.772 (*p* < 0.0001); DMDL rho = 0.666 (*p* < 0.0001); DL rho = 0.778 (*p* = 0.039); DM rho = 0.378 (*p* = 0.203); NNCD rho = 0.143 (*p* = 0.559).

**Discussion:**

Overall, and for the delirious subset, statistically significant agreement was present. Poor inter-test correlation existed in the groups without delirium (DM, NNCD).

**Conclusions:**

The MBTc correlates well with the MBTv in patients who are clinically suspected to have delirium but has poor correlation in patients without delirium. Visuospatial cognition and psychomotor deficits in a dementia cohort and mechanical factors (such as tremor, poor fingernail hygiene and visual impairment) in a group with no neurocognitive disorder may limit the utility of the MBTc in a hospitalised older population.

## Introduction

Delirium is estimated to occur in 21% of all inpatients [1] and is associated with longer inpatient stays and higher mortality rates [2]. Delirium is more prevalent in older patients (> 65 years old), especially in those with dementia [1], and is misdiagnosed, detected late or missed in over 50% of cases [3]. Not only is cognitive impairment a key element of delirium, it’s also a well-recognised risk factor for the development of the condition [4]. Impaired attention is the cardinal cognitive deficit associated with delirium and is a required feature for diagnosis according to criteria such as those outlined in the DSM 5 and ICD-10 [5, 6]. The months backwards test (MBT) is a brief, simple bedside test of attention that is also thought to engage short term memory, executive function and central processing [7, 8]. Bellelli et al. found that the MBT had a high sensitivity for delirium while Adamis et al. showed that the MBT was the single most sensitive test for identifying delirium in an elderly cohort (compared to digit span, vigilance A test, serial sevens) [9, 10]. Indeed, the MBT forms part of the test for attention in the Revised Delirium Rating Scale administration manual [11]. However, as evidenced by a systematic review of verbal MBT use, there are inconsistencies in its administration and interpretation [12]. The 22 articles in this review describe a variety of MBT metrics, the result of each decided and interpreted by a human assessor. The authors of this study propose that a human examiner may vary in test administration e.g. via error interpretation. A major challenge in detecting delirium is the lack of a convenient, universally applicable tool that assesses for impaired attention [13]. By employing computer technology, it is thought that inter-rater inconsistencies can be minimised [14]. We hypothesise that by having the test administered via a computer application, the test could be universally administered (by both experienced and inexperienced doctors, nursing staff and allied healthcare professionals) and interpreted with minimal need for prior training with reproducible results independent of the assessor. Given the evidence base already established (see above) for the verbal MBT, if a computerised version of the test correlated well with the verbal version that is administered by experienced and formally trained assessors, we hypothesise that the computerised MBT may be easier and more consistent to use for both experienced and unexperienced medical professionals alike.

In a preliminary study of a newly developed computerised months backwards test (application on a handheld android tablet; MBTc) comparing medically unwell elderly patients and cognitively intact young people, the MBTc had excellent sensitivity for detecting cognitive impairment [15]. This study found that the computerised MBT allows for accurate and efficient testing of general cognition in clinical populations. However, a limitation of this study was that dementia was only diagnosed in one patient; our study aims to explore MBTc use in this cohort. The aim of this pilot study is to observe if there is significant correlation between patient performance in the MBTc and the MBTv in a cohort with mixed neuropsychiatric conditions. We hypothesise that employing a standardised test on a handheld electronic tablet in an elderly cohort will allow ease of interpretation and reduce scoring inconsistencies for assessors of varying experience levels.

## Methods

Participants were recruited from the Psychiatry Of Old Age Consultation Liaison Services at Galway University Hospital (GUH) and Sligo University Hospital (SUH) between April 2015 and July 2017. All participants were over the age of 65.

The battery of tests applied included the revised Delirium Rating Scale (DRS-R98), Mini-Mental State Examination, Delirium Motor Subtype Scale, Informant Questionnaire of Cognitive Decline in the Elderly as well as standard clinical assessment, chart review and collateral history [11, 16–18]. Assessments were conducted by raters (MM, LL) specifically trained in the use of the tests included and, to further enhance inter-rater reliability, ratings associated with any uncertainty were discussed and agreed. Patients were assessed at the bedside during the late morning/early afternoon when the anchors of the day are thought to be optimally active. The traditional verbal assessment was completed first followed by the computerised version. Both tests were completed within approximately 10 min of each other. Delirium was diagnosed if one scored ≥ 15 in the DRS-R98 and/or met DSM IV criteria. Dementia was defined as a clear history of meeting DSM-IV criteria (based on all available information including clinical case notes and a collateral history) *or* Informant Questionnaire on Cognitive Decline in the elderly (IQCODE) score of ≥ 3.5. Comorbid delirium-dementia was defined as the presence of both disorders. Patients with neither delirium nor dementia were designated ‘No Neurocognitive disorder’ (NNCD).

Patients included were those over 65 years of age referred to the inpatient Psychiatry Liaison Service at GUH and SUH and were divided into the following groups: meeting criteria for delirium in the context of background dementia (DMDL), meeting criteria for delirium only (DL), meeting criteria for dementia (DM) and no neurocognitive diagnosis (NNCD). Patients excluded were unconscious/sedated patients (with a reduced Glasgow Coma Scale score), patients who were unable to attempt both bedside tests of attention and patients receiving care in the intensive care unit, high dependency unit and emergency department.

**Fig. 1:**
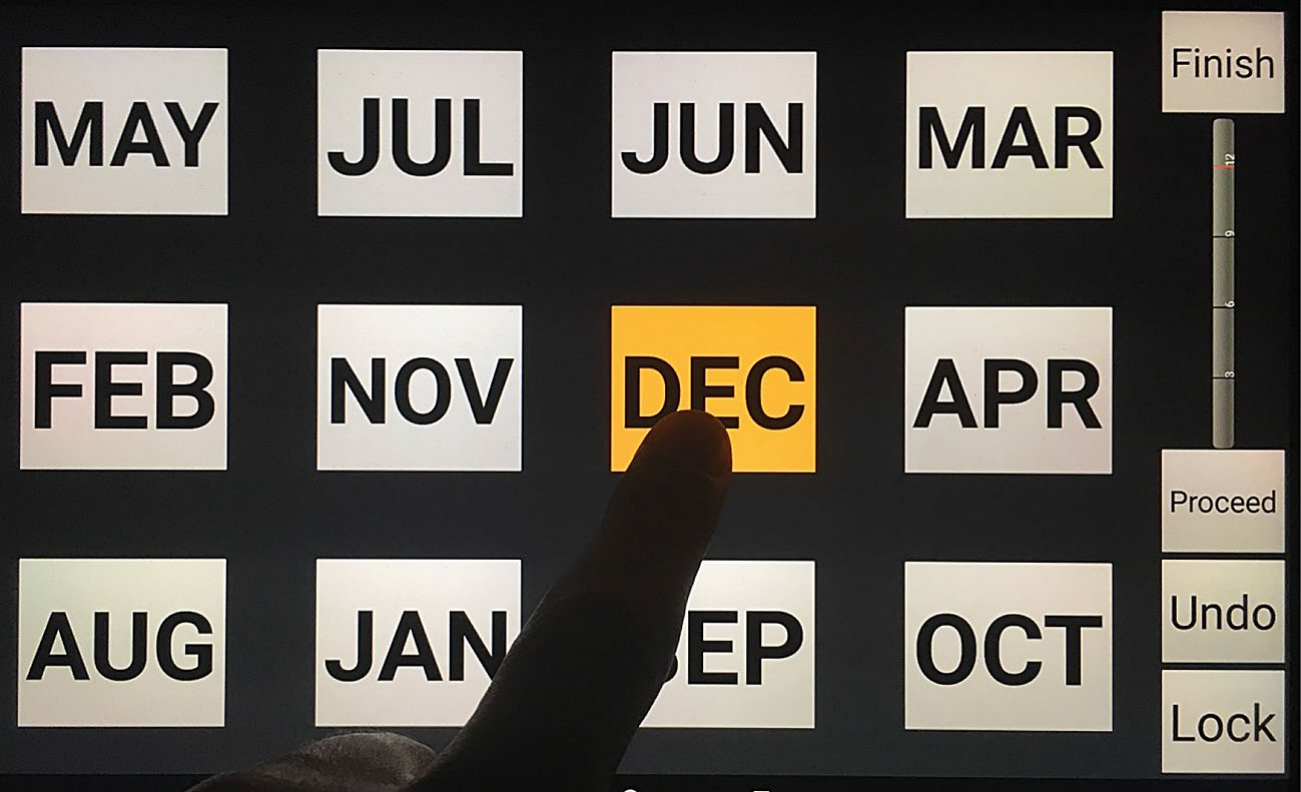
User-friendly app interface

A computerised version of the MBT and a verbal version of the MBT were administered. The application interface was designed to be user-friendly (See Fig. 1) presenting the twelve months on a 21 cm × 14 cm touch sensitive screen and asking the patient to touch the months in reverse order. The application month button design was modelled on the common abbreviated calendar appearance familiar to all age groups.

**Fig. 2:**
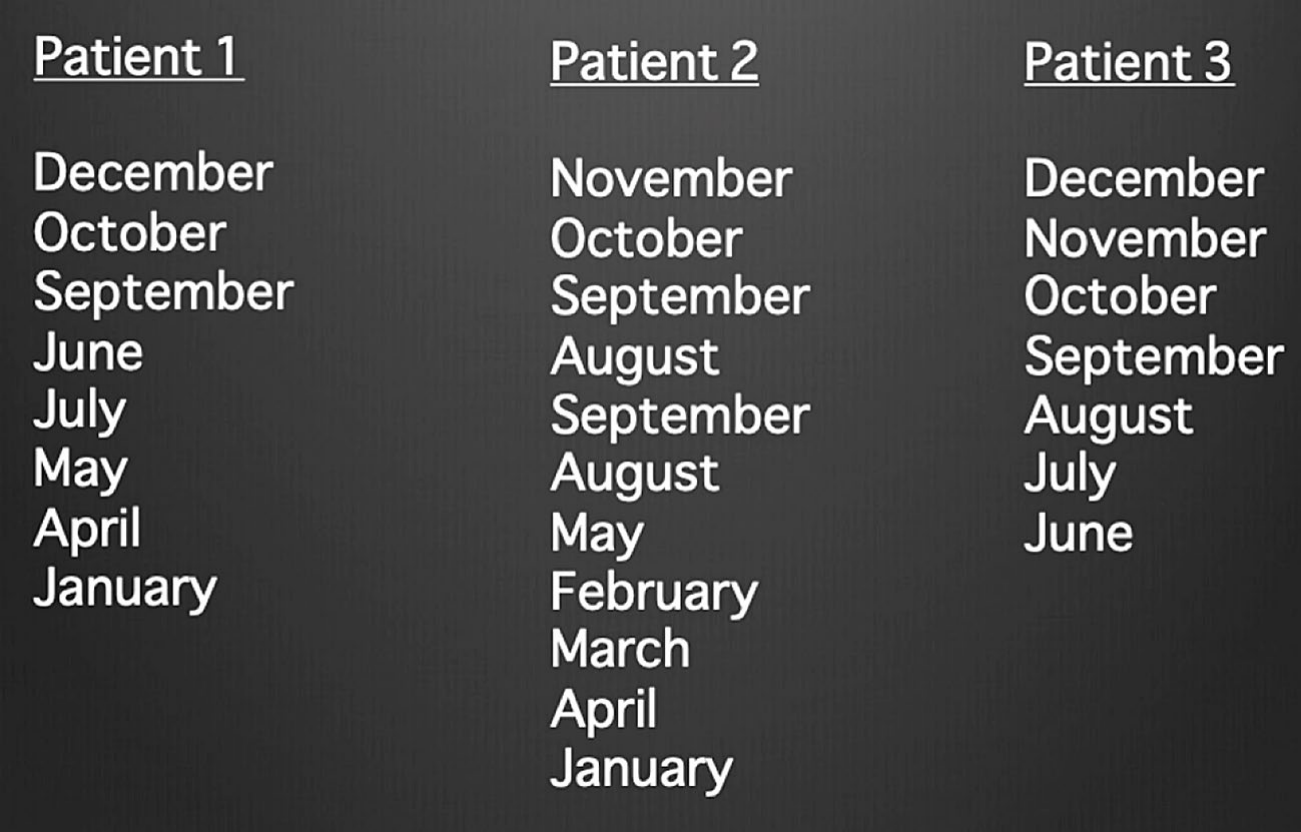
Sample answers demonstrating the potential variability in responses that require scoring

Due to the exponential number of possible answers (months skipped, months in an incorrect order, premature termination of attempt etc.), interpretation can be complex and requires a clear scoring system that removes inter-user and examiner variability (see varying examples in Fig. 2).

We employed a scoring algorithm to allow immediate and convenient interpretation of performance while reducing inter-user grading variability. A recent study identified that patients with delirium appeared to stop earlier in the reverse months progression than patients with dementia and this scoring system aims to take advantage of this differentiating characteristic [19]. The patient’s performance was assessed using a ‘Correct Progression’ scoring system: 1 point was allocated for correctly picking the final month first. Every correct progression after that was allocated 1 point. The patients were allocated a score out of 12 based on the above criteria. A cut-off of < 8 progressions was trialled in both (MBTv and MBTc) to separate inattention from attention. For example: Picking Dec. = 1 point; Dec. to Nov. = 1 point; Nov. to Oct. = 1 point; Sept. to Aug. = 1 point.

Statistical analysis was conducted using SPSS. The correlation between the MBTv and MBTc was analysed using Spearman’s rank correlation coefficient with the *p* < 0.05 used as the cut-off for statistical significance. Also, a Bland–Altman plot (scatter plot) was conducted to compare the two measures by plotting the score differences of MBTv and MBTc against their means. Because the two measures were not normally distributed, their natural logarithms were used (ln). In addition, two logistic regression analyses were used with vMBT and cMBT alternatively as predictors of delirium. In these regression analyses, the dependent variable was delirium vs. no delirium and the independent variables were the MBTv or MBTc, age, gender, MMSE score, and previous history of dementia. The Backward Stepwise Wald method was used.

## Results

### Demographic data

A total of 75 patients were recruited across both hospital sites. 30 patients were recruited from GUH and 45 patients from SUH. The gender split was as follows: 28 (37.3%) males, 47 (62.7%) females. The mean age of the cohort was 80.3 (SD = 7.61) years old. All patients assessed were able to attempt both bedside tests of attention.

### Cognitive subgroups based on clinical criteria

Patients were allocated a clinical diagnosis based on the aforementioned battery of clinical tests; Delirium Rating Scale (DRS-R98), Informant Questionnaire of Cognitive Decline in the Elderly (IQCODE) and DSM IV criteria for dementia and delirium (see also Methods section) and the final subgroup allocations were as follows: Thirty-six participants (*n* = 36 (48%)) were diagnosed with delirium in the context of an underlying dementia (DMDL), thirteen (*n* = 13 (17%)) were diagnosed with dementia only (DM), seven (*n* = 7 (9%)) were diagnosed with delirium only (DL), and nineteen (*n* = 19 (25%)) participants were considered to be cognitively intact (not meeting criteria for a diagnosis of delirium or dementia) (NCCD).

### Scales (MMSE, MBTv, MBTc, DMSS)

The mean MMSE of the total group was 20.4 (SD = 6.6). The mean MMSE scores together with mean age in each subgroup is shown in Table 1. Using a cut-off of < 8 progressions to separate inattention from attention (described in detail in the Methods section), the results comparing MBTv results with the MBTc results per subgroup are described.

**Table 1:** Summary table of mean age, mean MMSE, results from MBTv and MBTc per subgroup

Sub groups	Mean age (SD)	Mean MMSE (SD)	MBTv		MBTc	
			^a^Attention (*n*)	Inattention (*n*)	Attention (*n*)	Inattention (*n*)
DMDL	82 (8)	15 (6)	10	26	6	30
DM	79 (7)	21 (5)	12	1	10	3
DL	81 (9)	24 (5)	5	2	3	4
CI	79 (6)	28 (2)	19	0	17	2

Inattention (< 8 Progressions)

^a^Attention (= / > 8 Progressions)

Of the total sample, the mean MBTv was 8.1 (SD = 4.6) and the mean MBTc was 6.3 (SD = 4.4).

### Verbal and computerised test correlation

Given the evidence base for the verbal MBT, if a computerised version of the test correlated well with the verbal version administered by experienced and formally trained assessors, we hypothesise that the computerised MBT may be easier and more consistent to use for both experienced and unexperienced medical professionals alike. To assess this correlation, a Spearman’s correlation test (MBTv and MBTc were normally distributed) was used to calculate the inter-test correlation coefficient (rho). Overall, a statistically significant positive inter-test correlation between the MBTv and MBTc (*p* < 0.0001) was found. Similarly, statistically significant positive correlations were found for the groups of DMDL and DL group (*p* < 0.0001). However, poor correlation was found in the two groups without delirium DM and NNCD. (See also Table 2).

**Table 2:** Spearman correlation of MBTv with MBTc

	rho	*p*-value
Overall	0.772	< 0.0001
DMDL	0.666	< 0.0001
DL only	0.778	0.039
DM only	0.378	0.203
Cog intact	0.143	0.559

### Bland–Altman plot

**Fig.3:**
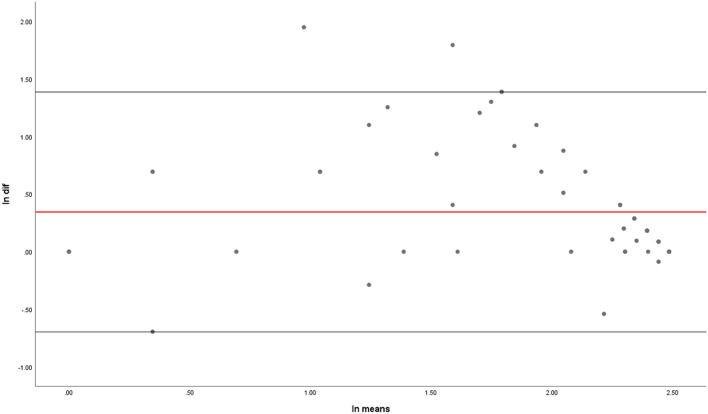
Bland–Altman plot

The Bland–Altman plot is depicted in Fig. 3

Figure 3 shows that the two methods are comparable (with the mean of difference being close to 0). It also shows systematic variation with the mean of the two measurements towards to the right which reflects more patients who were cognitively intact than those with previous history of dementia (as described above). The standarised Beta coefficient showed no significant differences between the two measurements (B = – 0.219, t = – 1.770, *p* = 0.082).

### Logistic regression

The results of the two logistic regression analysis perfomed for the MBTv and MBTc (cut-off point for both < 8) are illustrated in Tables 3, 4.

**Table 3:** Regression analysis including MBTv

	*B*	S.E	Wald	df	Sig	Exp (*B*)
MBTv (inattention)	2.589	1.183	4.791	1	0.029	13.313
MMSE score	– 0.174	0.076	5.316	1	0.021	0.840
Constant	3.411	1.807	3.565	1	0.059	30.309

**Table 4:** Regression analysis including MBTc

	*B*	S.E	Wald	df	Sig	Exp (*B*)
MBTc (inattention)	1.715	0.727	5.560	1	0.018	5.556
MMSE score	– 0.195	0.074	7.053	1	0.008	0.823
Constant	3.651	1.804	4.096	1	0.043	38.499

From the tables above seems that both MBTv and MBTc are significant predictors of delirium together with MMSE when controlling for other variables

## Discussion

These results imply very strong correlation between the MBTv (done by formally trained experienced assessors) with the MBTc both overall and in the subgroups meeting a clinical diagnosis of delirium (DMDL, DL). There is poor correlation in the dementia only group and in the group with no neurocognitive disorder (DM, NNCD). Given that poor attention is a cardinal feature of delirium and is required in both the verbal and computerised version, it is unsurprising that participants who met the clinical criteria for delirium did poorly in both. When reviewing the poor correlation between the verbal and computerised versions in the dementia group, more participants had less than 8 correct progressions in the computerised version than in the verbal version (3 vs 1; DM subgroup – see Table 1). Visuospatial cognition such as spatial orientation can be impaired in dementia and may partly account for the difference in completion of the computerised version in the dementia subgroup. Psychomotor deficits in group due to underlying neurodegeneration can also result in poor computerised performance [20]. Poor correlation between the verbal and computerised version also exists in the cohort with no neurocognitive disorder (NNCD). In the elderly hospitalised group with NNCD, practical limitations of the computerised tablet noted by the authors were the presence of a tremor (influencing month selection), visual difficulties (cataracts, diabetic retinopathy) and, surprisingly, length of fingernails (obstructing touchscreen utility). The explanation for the poor correlation between the MBTv and MBTc score is likely to be due to the reduced performance in the computerised test due to the aforementioned mechanisms. In addition, the distribution of subjects, high homogeneity and small sample sizes of the groups (in this pilot study) may be another reason for the low correlation of the two tests in the groups without delirium. Given that the correlation between tests was high when numbers were large in the entire sample, a larger follow-on study to this initial pilot study with larger sample sizes in each subgroup is needed to fully assess the utility of this tool.

A possible limitation of this study is that the oral battery of tests (as described previously), including the MBTv, was conducted before the patient attempted the MBTc version; this may have affected the performance in the latter (improved due to the possible benefit of a ‘verbal practice-run’ or disimproved due to patient fatigue); randomization and reversal of the chronology may be of interest in future studies to mitigate the effects of the previously described practice effect and patient fatigue.

Advantages of the tablet was that employment of an algorithm allowed instantaneous interpretation (relative to verbal interpretation) with no inter-user variability. The application’s easy-to-use interface does not require interpretation training and can be used by all health care professionals. A financial review identifying the cost–benefit of the purchase of a moderately priced handheld tablet use vs. reduction in the length of inpatient stay through early identification of deterioration (for example, if used on routine nursing rounds) would also help evaluation of its practical implementation.

As part of the Integrated Care Programme for Older Persons there is an emphasis on the role of integrated computer technology (ICT) in older persons care [21]. Given the visuospatial limitations in the dementia subgroup and the physical limitations in the group with no neurocognitive disorder (tremor, long fingernails) that our study has identified, one could envisage future development of an application based on advancing speech recognition technology that allows a would allow a hybrid voice recognition computerised months backwards test that would circumvent visuomotor problems yet have all the previously mentioned benefits of standardisation.

## Conclusions

The MBTc correlates well with the MBTv in patients who are clinically suspected to have delirium but has poor correlation in patients without delirium. Visuospatial cognition and psychomotor deficits in a dementia only cohort and mechanical factors (such as tremor, poor fingernail hygiene and visual impairment) in a group with no neurocognitive disorder may limit the utility of the MBTc in a hospitalised older population. A larger follow-on study to this pilot study is needed to fully evaluate its use.
